# Primary Biliary Cholangitis Increases Mortality Irrespective of Presence or Absence of Cirrhosis

**DOI:** 10.1111/liv.70610

**Published:** 2026-03-17

**Authors:** Lars Bossen, Henning Grønbæk, Peter Ott, Peter Jepsen

**Affiliations:** ^1^ Department of Hepatology & Gastroenterology Aarhus University Hospital Aarhus Denmark; ^2^ Department of Internal Medicine Horsens Regional Hospital Horsens Denmark; ^3^ Department of Clinical Epidemiology Aarhus University Hospital Aarhus Denmark

**Keywords:** cirrhosis, hepatocellular carcinoma, incidence, prevalence, primary biliary cholangitis, prognosis

## Abstract

**Background & Aims:**

Primary biliary cholangitis (PBC) is an autoimmune liver disease whose effect on long‐term survival remains unclear. We aimed to compare the prognosis of patients with biopsy‐confirmed PBC with population comparators.

**Methods:**

We used nationwide healthcare registries to include all Danish patients diagnosed with histologically confirmed PBC in 1998–2020. We estimated the prevalence of PBC and the incidence in 2016–2019. Dividing PBC by presence or absence of cirrhosis, we used the cumulative incidence function to estimate the risk of death and HCC. We included 5:1 age‐ and sex‐matched population comparators.

**Results:**

We included 1163 PBC patients (88.1% women, median age at diagnosis = 59.7 years); those with cirrhosis were older (median 63.9 vs. 59.0). Patients had more comorbidity at diagnosis than comparators, especially connective tissue disease. On January 1st 2021, the prevalence of PBC was 22.3 per 100,000 population. The incidence rate in 2016–2019 was 2.80 (95% CI: 2.58–3.02) per 100,000 population per year. PBC patients with cirrhosis had a higher 10‐year risk of death than their matched comparators, adjusted relative risk = 2.41 (95% CI: 1.89–3.09). For patients with non‐cirrhotic PBC, the 10‐year risk of death was 18.5% (95% CI: 15.5–21.7) versus 14.3% (95% CI: 13.0–15.6) for their comparators, adjusted relative risk = 1.22 (95% CI: 1.03–1.47). Patients with cirrhotic PBC had a 10‐year risk of HCC at 2.6% (95% CI: 0.8–6.0).

**Conclusions:**

Patients with PBC have a worse prognosis than the general population irrespective of the presence or absence of cirrhosis at the time of PBC diagnosis.

## Summary

Primary biliary cholangitis (PBC) is a rare chronic liver disease which can cause development of liver cirrhosis, but its impact on survival is poorly understood, particularly for those without liver cirrhosis. We compared mortality risk between Danish patients with PBC and the general population, matching them on sex and age. We found that patients with PBC without cirrhosis had a slightly higher mortality risk than the general population (18.5% vs. 14.3% after 10 years), but the difference may not be attributable to PBC alone.

**Abbreviations**
AIH: autoimmune hepatitis
CI: confidence interval
DNPR: Danish National Patient Registry
HCC: hepatocellular carcinoma
ICD‐10: international classification of diseases, 10th edition
IQR: interquartile range
PBC: primary biliary cholangitis
UDCA: ursodeoxycholic acid

## 1 Introduction

Primary biliary cholangitis (PBC) is a rare chronic autoimmune liver disease characterised by cholestasis and destruction of intrahepatic bile ducts ultimately resulting in liver fibrosis with progression to cirrhosis and possibly hepatocellular carcinoma (HCC) [1, 2, 3]. The histological stage is divided into stage 1–4 according to the Ludwig classification with stage 4 being cirrhosis [4]. Most patients are diagnosed with PBC before they develop cirrhosis [5]. There is a large geographical variation in the prevalence and incidence of PBC with the most recently presented pooled European prevalence around 14.6 per 100,000 population and incidence being 1.9 per 100,000 population per year [3].

The effect of PBC on long‐term survival remains uncertain. In 1999, Poupon et al. included 225 PBC patients at the beginning of ursodeoxycholic acid (UDCA) treatment and compared 10‐year survival with a standardised control cohort from the French population [6]. They showed that patients in a non‐cirrhotic stage had a 10‐year survival probability similar to that of a standardised control cohort, whereas patients with PBC in the cirrhotic stage had a reduced 10‐year survival probability [6]. More recently, Murillo Perez et al. showed that histological stage is a predictive marker of prognosis in PBC patients, using a large cohort of patients from the GLOBAL PBC study group [7]. This prognostic effect of fibrosis was supported by data from the UK‐PBC study group [8], yet none of the two studies compared mortality with the general population. A recent Swedish register‐based study compared survival in PBC patients with a reference cohort from the general population and showed an increased mortality in PBC patients; however, they did not investigate whether this was dependent on histological stage at diagnosis [9]. Further, the effects of PBC on HCC risk, too, depend on histological stage [10].

For this study we hypothesized that long‐term survival of patients with PBC depends on histological stage at PBC diagnosis, and that PBC patients have a higher risk of death and HCC than age‐ and sex‐matched comparators independently of the histological stage at diagnosis. Thus, we aimed to investigate the 1‐, 5‐, and 10‐year risk of death and 10‐year risk of HCC in patients with PBC, based on their histological stage at diagnosis and compared with age‐ and sex‐matched comparators from the general population.

## 2 Methods

### 2.1 Study Population

This is a cohort study based on nationwide registries including all Danish patients with biopsy‐verified PBC diagnosed in 1998–2020. The Danish Data Protection Agency approved the data collection from the registries. According to Danish law, no further ethical approval was required since it was carried out on data from registries.

### 2.2 Data Sources

In Denmark, all residents have access to the public tax‐funded healthcare system for diagnostic and therapeutic procedures, and for public hospitals it has been mandatory to report data on inpatient admissions (since 1977) and outpatient visits (since 1995) to the Danish National Patient Registry (DNPR) [11]. Data from the DNPR include dates of admission and discharge and diagnosis codes. Since 1994 diagnoses have been coded according to the International Classification of Diseases, 10th edition (ICD‐10). We used data from the Danish National Pathology Registry that stores information from histo‐pathological examinations using the SNOMED coding and has been nationwide since 1997 [12]. Through the Danish Cancer Registry, where all cancers are coded using ICD‐10 codes, we collected data on HCC diagnoses [13], and from the Danish Civil Registration System we obtained data on dates of death or emigration [14]. Further, we retrieved data from the Register of Pharmaceutical Sales to identify patients who had filled a prescription for UDCA [15]. We were able to link individual‐level data across the registries using the unique personal identification number given to all Danish citizens at birth or immigration. In this study we chose to include patients diagnosed with PBC after January 1st 1998 to ensure that patients were in fact newly diagnosed.

### 2.3 Inclusion Criteria

We used different inclusion criteria for follow‐up analyses and for analyses of incidence and prevalence. This was necessary because data on UDCA were only available from October 2014.

For the follow‐up analyses, we included all patients with a recorded diagnosis of PBC in both the DNPR and the Pathology Registry. This requirement for double registration was based on a preliminary analysis that convinced us that we would include patients without PBC if we included patients who were only registered in one of the registries (Figure S1 and Table S1). We used the date of the later of the two recorded diagnoses as the ‘index date’. With this approach we knew that we would exclude some patients with PBC from the study. Thus, for the analyses of prevalence and incidence we included all patients in the follow‐up analyses plus everybody with a recorded diagnosis of PBC in the DNPR who had also filled a prescription for UDCA. Here, we used the later of the date of diagnosis in the DNPR and the date of first collected UDCA prescription as the index date. Patients were excluded if they were liver transplanted before the index date.

### 2.4 Patients

In the DNPR we identified patients with the ICD‐10 code K743 (‘Primary biliary cirrhosis’) or K732E (‘Autoimmune hepatitis with concomitant primary biliary cirrhosis’) as their primary diagnosis code and patients with K743 as secondary diagnosis if they also had autoimmune hepatitis (AIH) as primary diagnosis (ICD‐10: “K732B”, “K732C”, “K732G” or “K754”). In the Pathology Registry we identified patients with the SNOMED code M49590 (“Primary biliary cirrhosis”), M49591 (‘Primary biliary cirrhosis, non‐cirrhotic stage’), and M49592 (‘Primary biliary cirrhosis, cirrhotic stage’). Further, we identified patients as having cirrhosis at diagnosis if they had a SNOMED code of cirrhosis before, or on the same day as, the index date. Likewise, if patients had any of the following ICD‐10 codes in the DNPR before their index date, they were considered to have cirrhosis at diagnosis; R18* (“Ascites”), K65.8I (‘Spontaneous bacterial peritonitis’), K76.7 (“Hepatorenal syndrome”), I85.0 or I86.4A (“Variceal bleeding”), K74.6 (‘Other and unspecified cirrhosis of liver’), or K72.1 (‘Chronic hepatic failure’). If patients had unknown histological stage in the Pathology Registry (i.e., the SNOMED code M49590 (‘Primary biliary cirrhosis’)) and did not fulfil any of the listed criteria for having cirrhosis, they were considered not to have cirrhosis at diagnosis. Liver transplantation was identified in the DNPR using the ICD‐10 code Z944 (‘Liver transplantation’). We used the ICD‐10 code C22.0 to identify HCCs in the Cancer Registry (1998–2018) and DNPR (2018–2020). UDCA was identified using the ATC code A05AA02.

### 2.5 Comparators

We included 5 age‐ and sex‐matched comparators from the general Danish population per PBC patient. These comparators had to be alive and without PBC on the index date of their corresponding PBC patient. We used the Charlson Comorbidity Index to identify comorbid diseases in the DNPR. This index contains 19 weighted disease domains that sum a total score of comorbidity [16, 17], but in this study we did not include liver disease in the calculation of the comorbidity score.

### 2.6 Statistical Analyses

We calculated the prevalence of PBC in Denmark on January 1st 2021 as the number of patients alive with PBC divided by the total number of citizens in Denmark. We computed an overall incidence rate of PBC in 2016–2019 as well as yearly incidence rates by dividing the number of new PBC patients by the total number of Danish citizens at the beginning of each year 2016–2019. The incidence rates were age‐ and sex‐standardised to the Danish population on January 1st 2021 using direct standardisation. The demographics of the Danish population were retrieved from Statistics Denmark (www.statistikbanken.dk) (Table S2). We chose the time period 2016–2019 for incidence rate calculation to make sure we only included incident patients; we expected all previously diagnosed patients to have picked up UDCA at least once in the time period from mid‐October 2014 to end 2015.

In our follow‐up analysis, follow‐up started at the index date and patients were followed until death, emigration, liver transplantation or December 31st 2020. We used the cumulative incidence function to calculate the 1‐, 5‐, and 10‐year cumulative risk of death without liver transplantation for PBC patients with and without cirrhosis at diagnosis and for their respective controls treating liver transplantation as a competing risk [18]. Further, we estimated relative risks at 1, 5, and 10‐years adjusted for confounding by comorbidity diagnosed before the index date. This was done using the ‘pseudo‐observations’ method that allows confounder‐adjusted estimates of relative risk in the presence of competing risks [19]. The Charlson Comorbidity Index was included as a continuous variable. In Denmark, it is a relative contraindication to perform liver transplantation in patients older than 65 years. Therefore, we conducted another analysis to estimate the cumulative risk of liver transplantation in patients diagnosed before age 65. In this analysis, patients were followed from the index date until liver transplantation, turning 65 years (a competing risk‐event), dying before age 65 years without having received a liver transplant (a competing risk‐event), or censoring on December 31st 2020.

In our HCC analysis, patients and controls were followed from the index date until HCC, death without HCC, liver transplantation, emigration, or December 31st 2020. We used the cumulative incidence function to calculate the 10‐year risk of HCC, treating death without HCC and liver transplantation as competing risks. Moreover, in patients without cirrhosis at diagnosis and their matched comparators, we used the cumulative incidence function to calculate the 10‐year risk of developing cirrhosis while treating death as a competing risk.

We conducted sensitivity analyses: First, we estimated the 10‐year risk of death without liver transplantation in patients without cirrhosis at diagnosis while excluding those with unknown histological stage at diagnosis (unclassified in the Pathology Registry = SNOMED code: M49590, ‘primary biliary cirrhosis’) from this group to investigate if this exclusion changed the outcomes for this group. Second, we repeated our survival analysis after excluding patients with AIH overlap at diagnosis and their matched comparators, as patients with AIH overlap have a worse prognosis than PBC patients with no overlap [20]. Third, we estimated the survival without treating liver transplantation as a competing risk. Fourth, we estimated transplant‐free survival, that is, we considered liver transplantation or death as a composite endpoint. Fifth, we repeated our survival analysis, but moved the start of follow‐up to 1 year after the diagnosis. Some patients may have been diagnosed with PBC as part of a work‐up for another diagnosis with a really bad prognosis, that is, with a high short‐term mortality. In this fourth analysis, they were excluded from the analysis if they died within the first year after diagnosis.

## 3 Results

**FIGURE 1:**
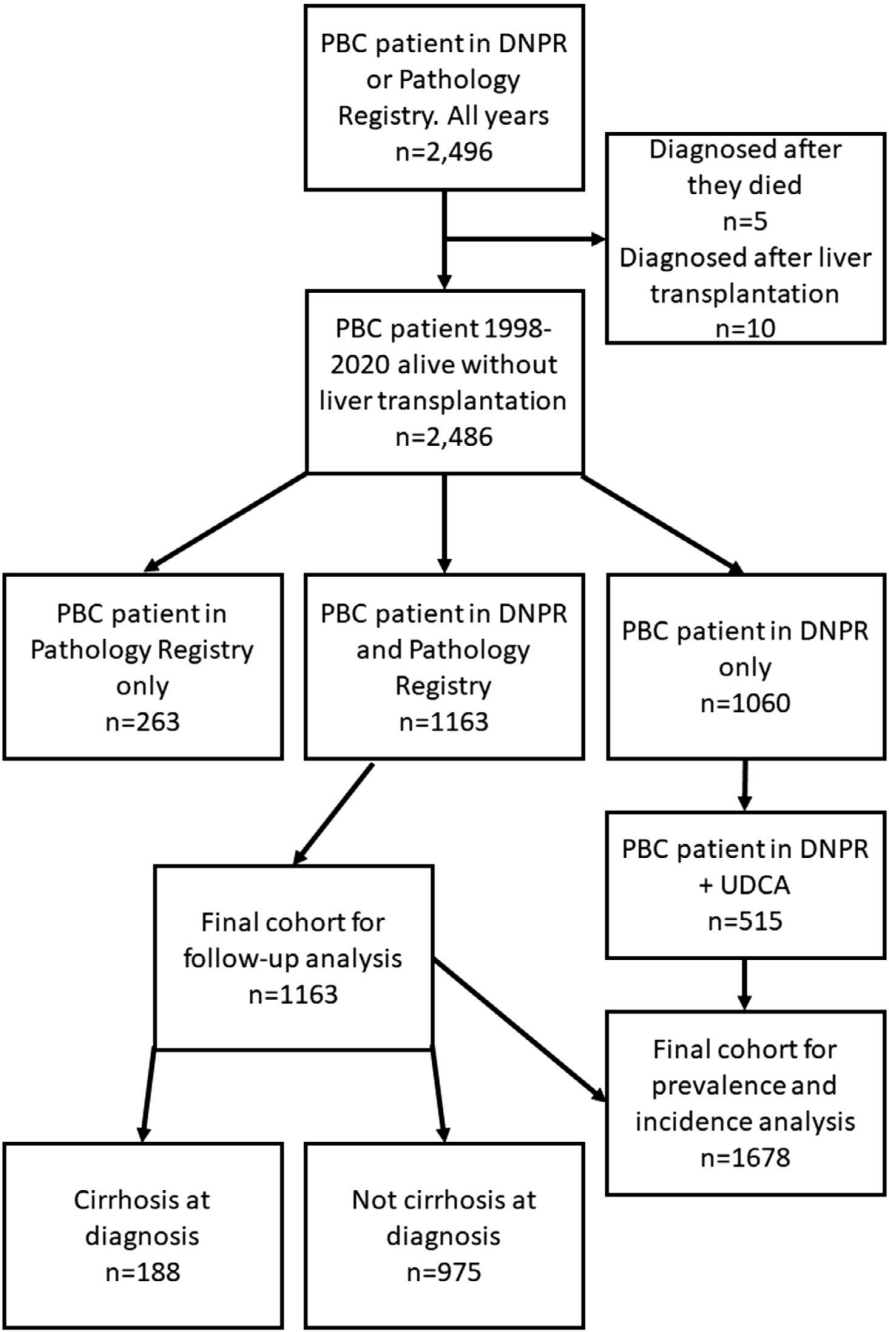
Flowchart of included patients. A total of 1163 patients with known histological stage at diagnosis were included in the follow‐up analysis and 1678 were included for prevalence and incidence. DNPR, Danish National Patient Registry; PBC, primary biliary cholangitis; UDCA, Ursodeoxycholic acid.

We identified a total of 2496 patients with PBC in either the DNPR or the Pathology Registry in 1998–2020 (Figure 1). A total of 1163 PBC patients with a PBC diagnosis recorded in both registries were included in the follow‐up cohort of whom 88.1% were women, median age at diagnosis was 59.7 years (IQR: 50.3–68.2) and 104 (8.9%) had AIH overlap. Of these 1163 patients included, 188 (16.2%) had cirrhosis at diagnosis while 975 (83.8%) did not (Figure 1, Table 1).

**TABLE 1:** Baseline characteristics of PBC patients based on their histological stage at diagnosis (cirrhosis or not).

	Cirrhosis patients	Comparators to cirrhosis patients
Number	188	936
Age at diagnosis, years, median (IQR)	63.9 (56.1–72.0)	63.7 (56.2–71.4)
Female sex, *n*(%)	161 (85.6)	794 (84.8)
Charlson Comorbidity Index score, % (0/1–2/3+)	63.8/28.7/7.5	74.9/21.4/3.7

	Non‐cirrhosis patients	Comparators to non‐cirrhosis patients
Number	975	4824
Age at diagnosis, years, median (IQR)	59.0 (49.3–67.2)	58.9 (49.2–67.0)
Female sex, *n*(%)	863 (88.5)	34 276 (88.6)
Charlson Comorbidity Index score, % (0/1–2/3+)	64.7/29.6/5.6	77.1/19.9/2.9

### 3.1 Prevalence and Incidence

In addition to the 1163 patients from the follow‐up analyses, we included 515 PBC patients from the DNPR who used UDCA (Figure 1). Some died or received a liver transplant before January 1st 2021, so on that date the cohort consisted of 1305 PBC patients, corresponding to a prevalence of 22.3 per 100,000 population. The overall incidence rate in 2016–2019 was 2.80 (95% CI: 2.58–3.02) per 100,000 population per year, varying between 2.00 (95% CI: 1.64–2.37) in 2018 and 3.47 (95% CI: 2.98–3.95) in 2019.

### 3.2 Prognosis

During a total follow‐up time of 8676 years (median follow‐up: 5.7 years), 265 of the 1163 PBC patients died, 47 were transplanted and four emigrated. The 10‐year cumulative risk of death before liver transplantation was 23.7% (95% CI: 20.7–26.8), and the 10‐year risk of liver transplantation was 4.7% (95% CI: 3.4–6.4), meaning that the 10‐year risk of death or liver transplantation was 28.4%. The 1163 patients and their 5760 matched comparators were included in the analyses of all‐cause mortality and HCC risk. PBC patients with cirrhosis at diagnosis were 4.9 years older than PBC patients without cirrhosis at diagnosis, and both PBC patients with and without cirrhosis had more comorbidity at diagnosis than their matched comparators (Table 1). The main difference in comorbidity at diagnoses came from connective tissue disease that was present in approximately 11% of patients and 3.0% in comparators (Table S3). Patients with PBC had a higher prevalence of peptic ulcer and chronic pulmonary disease than comparators, too. Among PBC patients, 2.1% of those with cirrhosis and 1.6% of those without cirrhosis had diabetes with end organ damage vs. 1.3% of comparators. All comorbidities at diagnosis are listed in Table S3.

**FIGURE 2:**
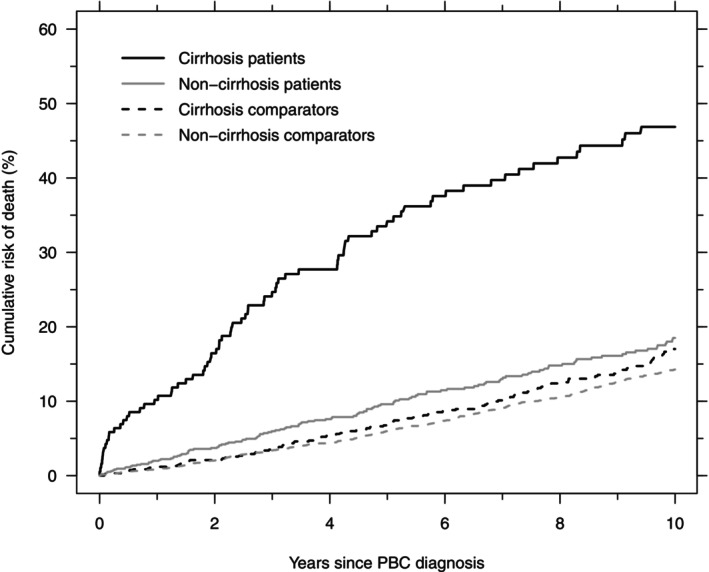
Cumulative risk of death in PBC patients with cirrhosis at diagnosis (black, solid), their comparators (black, dashed), PBC patients without cirrhosis at diagnosis (grey, solid), and their comparators (grey, dashed).

The 1‐, 5‐, and 10‐year cumulative risk of death before liver transplantation was higher in PBC patients diagnosed with cirrhosis than in their age‐ and sex‐matched comparators (Figure 2, Table 2). The adjusted relative risk of death before liver transplantation decreased from approximately 4 after 1 and 5 years to 2.4 after 10 years. Likewise, the 1‐, 5‐, and 10‐year cumulative risk of death before liver transplantation was higher in PBC patients diagnosed in a non‐cirrhotic stage than in their age‐ and sex‐matched comparators; 10‐year cumulative risk = 18.5% (95% CI: 15.5–21.7) and 14.3% (95% CI: 13.0–15.6), respectively (Figure 2, Table 2). The adjusted relative risk of death before liver transplantation dropped from 1.83 after 1 year to 1.22 after 10 years (Table 2).

**TABLE 2:** Risk of death before liver transplantation for PBC patients with and without cirrhosis at diagnosis and their respective comparators. 95% confidence intervals in parentheses.

	Cirrhosis patients	Comparators to cirrhosis patients	Relative risk (95% CI)^a^
1‐year risk of death	10.2% (6.4–15.0)	1.2% (0.6–2.1)	4.10 (1.09–15.5)
5‐ year risk of death	34.2% (27.1–41.3)	6.8% (5.2–8.6)	4.10 (2.87–5.86)
10‐year risk of death	46.9% (38.8–54.5)	17.0% (14.3–20.3)	2.41 (1.89–3.09)
10‐year risk of liver transplantation	8.4% (4.8–13.2)	—	—

	Non‐cirrhosis patients	Comparators to non‐cirrhosis patients	Relative risk (95% CI)^a^
1‐year risk of death	2.0% (1.2–3.0)	0.9% (0.6–1.2)	1.83 (0.79–4.24)
5‐year risk of death	9.6% (7.7–11.8)	6.0% (5.2–6.8)	1.51 (1.17–1.96)
10‐year risk of death	18.5% (15.5–21.7)	14.3% (13.0–15.6)	1.22 (1.03–1.47)
10‐year risk of liver transplantation	4.0% (2.6–5.9)	—	—
10‐year risk of cirrhosis	11.2% (8.8–13.9)	0.8% (0.5–1.1)	15.5 (9.34–25.7)

^a^ Adjusted for comorbidity at diagnosis.

Patients diagnosed with cirrhosis had a 10‐year risk of liver transplantation of 8.4% (95% CI: 4.8–13.2); it was 4.0% (95% CI: 2.6–5.9) for those diagnosed without cirrhosis, and no comparators were liver transplanted (Table 2). After excluding patients above age 65 from the analysis, the 10‐year risk of liver transplantation before age 65 years increased to 12.6% in cirrhosis patients and 5.5% in non‐cirrhosis patients. Further, PBC patients without cirrhosis at diagnosis had an 11.2% risk of developing cirrhosis within 10 years (Table 2).

During follow‐up, 13 PBC patients were diagnosed with HCC: 7 had cirrhosis from inclusion, of whom 1 was male, and of the 6 others, 3 were male. Three of those 6 were diagnosed with cirrhosis around the same time as their HCC diagnosis while the remaining 3 did not have cirrhosis. The 10‐year cumulative risk of HCC was highest in those diagnosed with cirrhosis from inclusion; 2.6% (95% CI: 0.8–6.0). Male patients had a higher risk of HCC than female patients both with and without cirrhosis.

### 3.3 Sensitivity Analyses

After excluding the 281 patients with unknown histological stage from the group of PBC patients without cirrhosis at diagnosis, the 10‐year mortality without liver transplantation was 19.6% (vs 18.5% in the primary analysis).

**FIGURE 3:**
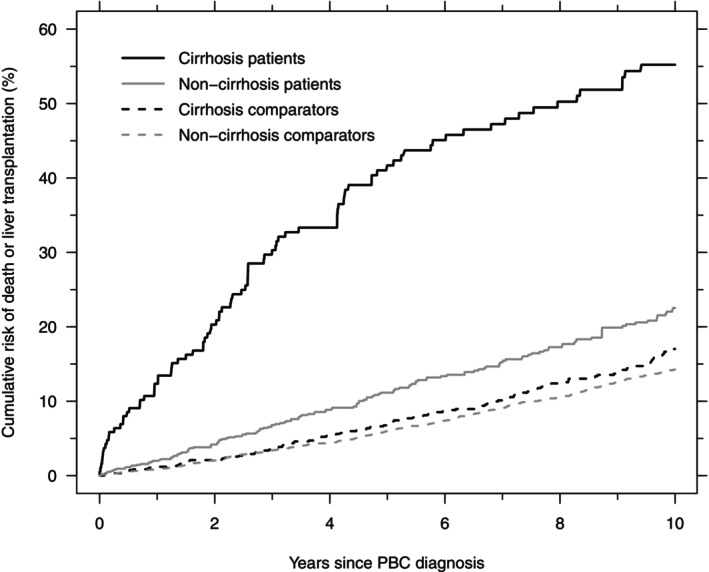
Cumulative risk of death or liver transplantation in PBC patients with cirrhosis at diagnosis (black, solid), their comparators (black, dashed), PBC patients without cirrhosis at diagnosis (grey, solid), and their comparators (grey, dashed).

Excluding patients with AIH at diagnosis and their matched comparators did not change the results. If not treating liver transplantation as a competing risk, the 10‐year mortality was 49.5% in cirrhosis patients (vs. 46.9% in the primary analysis), 17.0% in their comparators, 18.9% in non‐cirrhosis patients (vs. 18.5% in the primary analysis), and 14.4% in their comparators. If, instead, we considered liver transplantation or death as a composite endpoint, the 10‐year transplantation‐or‐mortality‐risk was 55.2% in cirrhosis patients and 22.5% in non‐cirrhosis patients (Figure 3). This analysis thus emphasised the long‐term consequences of PBC, even in the non‐cirrhotic stage.

When moving the start of follow‐up to 1 year after the date of diagnosis the adjusted relative risk of death was 3.51 in cirrhosis patients vs. their comparators after 5 years and 2.66 after 10 years (vs. 4.10 and 2.41, respectively, in the primary analysis). In non‐cirrhosis patients it was 1.47 after 5 years and 1.16 after 10 years vs. their comparators (vs. 1.51 and 1.22, respectively, in the primary analysis). Thus, our sensitivity analysis indicated that our findings were robust to the decisions we made in designing this study.

## 4 Discussion

In this nationwide population‐based cohort study we observed a prevalence of PBC on January 1st 2021 of 22.3 per 100,000 population. The overall incidence rate in 2016–2019 was 2.80 (95% CI: 2.58–3.02) per 100,000 population per year. Further, PBC patients had a worse prognosis than age‐ and sex‐matched comparators irrespective of the presence or absence of cirrhosis at the time of PBC diagnosis. Patients diagnosed with non‐cirrhotic PBC had a relative risk of death of 1.8 after 1 year, 1.5 after 5 years and 1.2 after 10 years compared with matched comparators. Those diagnosed with cirrhotic PBC had a higher relative risk of death compared with matched comparators: 4.1‐fold higher after 1 and 5 years, and 2.4‐fold higher after 10 years. The 10‐year risk of HCC for patients with cirrhotic PBC was 2.6%.

Our reported prevalence and incidence estimates are slightly higher than the pooled European estimates presented by Lv et al. in [3], but fit perfectly with the pooled prevalence (22.5 per 100,000 population) and incidence (2.35 per 100 000 population per year) from northern Europe published since 2000 [3]. Thus, our estimates of prevalence and incidence in Denmark did not deviate from similar populations.

A highly important finding in our study is the increased mortality in patients diagnosed with non‐cirrhotic PBC. This is in contrast with the French 1999 study by Poupon et al. who found a similar 10‐year survival in PBC patients without cirrhosis and matched controls [6]. Surprisingly, when comparing our survival estimates with their survival curves, our PBC patients without cirrhosis have a worse prognosis than theirs, whereas there is no obvious difference in the survival of the included comparators. The most likely explanation for the difference in survival among PBC patients is that they included a more selected cohort of PBC patients with a better survival than the average PBC patient, as more than 50% was included in a clinical trial of UDCA. This is in contrast to our study using population‐based nationwide registries to identify PBC patients. We cannot exclude the possibility that we included patients with a worse survival than the average PBC patient, but our preliminary analysis showed that our decision to use double registration minimised this problem. Further, the patients in this study were older than in the French study, and the time periods for the studies are non‐overlapping. Moreover, they did not include patients with AIH overlap, but according to our sensitivity analysis, excluding such patients should not affect the survival estimate. Thus, we believe that the apparent poorer survival for Danish vs. French patients with non‐cirrhotic PBC is that the French study included a selected group of non‐cirrhotic PBC patients: patients fit for a randomised trial of UDCA treatment.

A related discrepancy with the French Poupon study is that we found—unlike Poupon—that patients with non‐cirrhotic PBC had a poorer survival than the matched population comparators. It is possible that this poorer survival is due to uncontrolled confounding rather than to PBC itself. We matched on age and sex—two major confounders for prognosis, and we adjusted for comorbidity at diagnosis to account for the excess comorbidity observed in PBC patients at diagnosis. A possible uncontrolled confounder is thyroid disease as this is associated with PBC and, if uncontrolled, has a worse survival [21], yet a 2017 paper found that thyroid disease in PBC did not affect the rate of hepatic complications or the natural history of PBC [22]. Further, we did not control for socioeconomic status that may be a confounder in this study, as low socioeconomic status is associated with poorer survival. The association between socioeconomic status and PBC is, however, not as evident, but PBC is associated with smoking and clusters in urban areas with a history of coal mining industry that may be markers of a lower socioeconomic status [23, 24]. In 2016, a study found somewhat divergent results when investigating the association between socioeconomic status and PBC as they found that PBC incidence increased with increasing national human development index, but decreased with increasing education index [25]. Taken together, we do not think uncontrolled confounding significantly affected our conclusions, and we believe that PBC patients diagnosed without cirrhosis do have a worse prognosis than matched comparators from the general population. We do not think this conclusion conflicts with the Poupon paper because, as mentioned above, the difference most likely stem from different populations of non‐cirrhotic PBC patients, with their patients being more selective with a better prognosis.

A possible bias introduced by the decision that patients must be recorded in two registries in our follow‐up analysis is that we may have excluded some patients who truly had PBC. The diagnostic criteria for PBC no longer require a biopsy [2], so it is possible that our cohort is biased towards complex or atypical cases. We believe that any such bias will be small, however, because during the study period it was standard practice in many Danish centres to perform a biopsy in PBC patients at diagnosis to ascertain the histological stage. It is unknown why some patients with (suspected) PBC did not have a biopsy performed, and the criteria may vary from hospital to hospital. Our preliminary analysis showed that patients diagnosed only in the DNPR had a worse prognosis than doubly registered patients (10‐year mortality without liver transplantation = 44.2% vs. 23.7%), suggesting that many of the first did in fact not have PBC, but some other disease with a stronger negative impact on survival. This suggestion is further corroborated by the different female to male ratios in the cohorts, and by the different proportions of patients with an alcohol‐related diagnosis (7.2% vs. 1.6% in those from both registries) (Table S1). Yet, we cannot rule out that those patients with a true PBC diagnosis in DNPR but without biopsy actually had more severe PBC disease; that is a limitation of our study. However, the possibility that we excluded some of the sicker PBC patients cannot change our main finding that PBC patients diagnosed without cirrhosis have a worse prognosis than matched comparators, and from a clinician's point of view, it is more likely that we excluded some of the *healthier*, more typical PBC patients by insisting on biopsy confirmation. Despite these considerations about the direction of selection bias, we see it as a strong point of our study that patients were recorded in both registries with a high certainty of the PBC diagnosis.

Patients with non‐cirrhotic PBC had higher 10‐year mortality than their matched comparators, but the difference was small, 18.5% vs. 14.3%. The clinical significance of this difference can be questioned, and it cannot be attributed to PBC alone: The relative risk of death was slightly larger when follow‐up began at PBC diagnosis than when it began 1 year later (1.22 vs. 1.16 after 10 years), suggesting that comorbid conditions that led to the PBC diagnosis may have contributed to a higher mortality in the first year after PBC diagnosis. Moreover, the uncertainty concerning the direction and extent of selection bias discussed in the previous paragraph calls for caution in the interpretation of these differences in mortality.

Another limitation to our study is the low number of HCCs observed. With this low number of events, our estimates were imprecise, and we were unable to investigate risk factors for HCC development other than sex and presence or absence of cirrhosis.

In conclusion, we have shown that the prevalence and incidence of PBC in Denmark are similar to estimates from northern Europe published after year 2000. Further, we have shown that 55.2% of PBC patients with cirrhosis and 22.5% of PBC diagnosed without cirrhosis are transplanted or die within 10 years of their diagnosis, and that the risk of death is higher than in age‐ and sex‐matched comparators, even in patients diagnosed before they develop cirrhosis. However, the excess mortality for patients with non‐cirrhotic PBC was small and possibly not caused by PBC alone. These findings strengthen our understanding of the clinical course of PBC, and they will likely be considered pertinent by patients with PBC and their caregivers.

## Author Contributions

L.B., H.G. and P.J. designed the study. L.B. collected the data and performed the statistical analysis. L.B. and P.J. interpreted the data. L.B. wrote the manuscript. All authors commented on the manuscript and accepted the final draft.

## Funding

L.B. and H.G. have received an investigator‐initiated research grant from Intercept. L.B. also received funding from Knud og Edith Eriksens Mindefond.

## Ethics Statement

According to Danish law, studies based entirely on data from healthcare registers—such as our study—need neither ethics approval nor patient consent.

## Conflicts of Interest

L.B. and H.G. have received an investigator‐initiated research grant from Intercept. H.G. also received research grants from Abbvie, Arla, ADS AIPHIA Development Services AG, and the NOVO Nordisk Foundation.

## Supporting information


**Figure S1:** Cumulative risk of death in PBC patients diagnosed in the DNPR and the Pathology Registry (black), patients diagnosed in the DNPR only (blue), and patients diagnosed in the Pathology Registry only (red). The 10‐year cumulative risk of death before liver transplantation was 44.2% (95% CI: 40.1–48.2) in those in the DNPR only, with a relative risk of 1.87 (95% CI: 1.58–2.20) vs. those diagnosed in both the DNPR and the Pathology Registry (10‐year risk = 23.7% (95% CI: 20.7–26.8)). In those diagnosed only in the Pathology Registry it was 34.2% (95% CI: 27.4–41.2) with a relative risk of 1.48 (95% CI: 1.14–1.91) vs. those diagnosed in both registries.
**Table S1:** Baseline characteristics of all identified patients in 1998–2020 in either of the two registries.
**Table S2:** The Danish population on January 1st 2021 used for direct standardisation.
**Table S3:** Comorbidities from the Charlson Comorbidity Index in patients and comparators at diagnosis. Numbers (%). Sorted by prevalence among PBC patients with cirrhosis at diagnosis.

## Data Availability

The data that support the findings of this study are available from The Danish Health Data Authority. Restrictions apply to the availability of these data, which were used under license for this study. Data are available from https://english.sundhedsdatastyrelsen.dk/health%E2%80%90data%E2%80%90and%E2%80%90registers with the permission of The Danish Health Data Authority.
