# Supplementary material for: Primary Biliary Cholangitis Increases Mortality Irrespective of Presence or Absence of Cirrhosis
Source: Liver Int. 2026 Mar 17;46(4):e70610. doi: 10.1111/liv.70610 (PMC12996748; doi:10.1111/liv.70610)
Supplement: Supplementary file 1 — Figure S1: Cumulative risk of death in PBC patients diagnosed in the DNPR and the Pathology Registry (black), patients diagnosed in the DNPR only (blue), and patients diagnosed in the Pathology Registry only (red). The 10‐year cumulative risk of death before liver transplantation was 44.2% (95% CI: 40.1–48.2) in those in the DNPR only, with a relative risk of 1.87 (95% CI: 1.58–2.20) vs. those diagnosed in both the DNPR and the Pathology Registry (10‐year risk = 23.7% (95% CI: 20.7–26.8)). In those diagnosed only in the Pathology Registry it was 34.2% (95% CI: 27.4–41.2) with a relative risk of 1.48 (95% CI: 1.14–1.91) vs. those diagnosed in both registries. Table S1: Baseline characteristics of all identified patients in 1998–2020 in either of the two registries. Table S2: The Danish population on January 1st 2021 used for direct standardisation. Table S3: Comorbidities from the Charlson Comorbidity Index in patients and comparators at diagnosis. Numbers (%). Sorted by prevalence among PBC patients with cirrhosis at diagnosis. [file LIV-46-0-s001.docx]

**Primary biliary cholangitis increases mortality irrespective of presence or absence of cirrhosis**

**Supplementary Figure 1:** Cumulative risk of death in PBC patients diagnosed in the DNPR and the Pathology Registry (black), patients diagnosed in the DNPR only (blue), and patients diagnosed in the Pathology Registry only (red). The 10-year cumulative risk of death before liver transplantation was 44.2% (95% CI: 40.1-48.2) in those in the DNPR only, with a relative risk of 1.87 (95% CI: 1.58-2.20) vs. those diagnosed in both the DNPR and the Pathology Registry (10-year risk = 23.7% (95% CI: 20.7-26.8)). In those diagnosed only in the Pathology Registry it was 34.2% (95% CI: 27.4-41.2) with a relative risk of 1.48 (95% CI: 1.14-1.91) vs. those diagnosed in both registries.


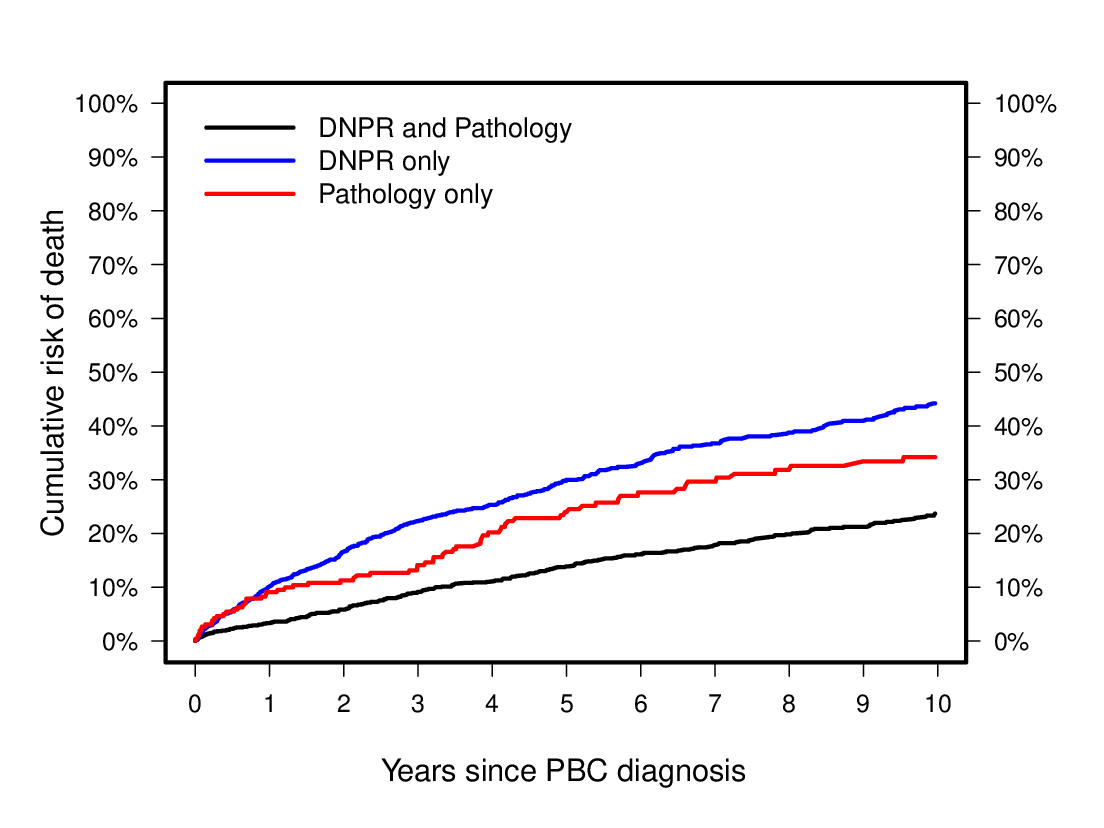


**Supplementary Table 1:** Baseline characteristics of all identified patients in 1998-2020 in either of the two registries.

|  | **DNPR and Pathology Registry** | **DNPR only** | **Pathology registry only** |
| --- | --- | --- | --- |
| Number of patients | 1163 | 1060 | 263 |
| Age at diagnosis, years, median (IQR) | 59.7 (50.3-68.2) | 64.4 (54.4-74.1) | 58.5 (46.8-69.8) |
| Female gender, n (%) | 1024 (88.1) | 850 (80.2) | 200 (76.1) |
| AIH overlap at diagnosis, n (%) | 104 (8.9) | 56 (5.3) | 29 (11.0) |
| Alcohol diagnosis*, ever, n (%) | 19 (1.6) | 76 (7.2) | 5 (1.9) |

*ICD-10 codes: “DK70*”

**Supplementary Table 2:** The Danish population on January 1^st^ 2021 used for direct standardisation.

| **Gender** | **Age** | **Population** |
| --- | --- | --- |
| Female | 0-29 | 1010065 |
|  | 30-39 | 342505 |
|  | 40-49 | 374191 |
|  | 50-59 | 398889 |
|  | 60-69 | 338832 |
|  | 70-79 | 301943 |
|  | 80-89 | 136546 |
|  | 90+ | 32217 |
|  |  |  |
| Male | 0-29 | 1058925 |
|  | 30-39 | 354174 |
|  | 40-49 | 374633 |
|  | 50-59 | 402277 |
|  | 60-69 | 328751 |
|  | 70-79 | 272754 |
|  | 80-89 | 100102 |
|  | 90+ | 13241 |

**Supplementary table 3:** Comorbidities from the Charlson Comorbidity Index in patients and comparators at diagnosis. Numbers (%). Sorted by prevalence among PBC patients with cirrhosis at diagnosis.

|  | **Cirrhosis patients** | **Cirrhosis comparators** | **Non-cirrhosis patients** | **Non-cirrhosis comparators** |
| --- | --- | --- | --- | --- |
| Connective tissue disease | 20 (10.6) | 27 (2.9) | 110 (11.3) | 126 (2.6) |
| Cerebrovascular disease | 17 (9.0) | 111 (11.9) | 102 (10.5) | 427 (8.9) |
| Chronic pulmonary disease | 15 (8.0) | 49 (5.2) | 72 (7.4) | 259 (5.4) |
| Ulcer disease | 14 (7.5) | 30 (3.2) | 39 (4.0) | 81 (1.7) |
| Any tumour | 14 (7.5) | 75 (8.0) | 82 (8.4) | 407 (8.4) |
| Diabetes I and II | 8 (4.3) | 10 (1.1) | 32 (3.3) | 72 (1.5) |
| Myocardial infarction | 5 (2.7) | 31 (3.3) | 18 (1.9) | 99 (2.1) |
| Peripheral vascular disease | 5 (2.7) | 23 (2.5) | 42 (4.3) | 102 (2.1) |
| Diabetes with end organ damage | 4 (2.1) | 12 (1.3) | 16 (1.6) | 61 (1.3) |
| Moderate to severe kidney disease | 3 (1.6) | 12 (1.3) | 12 (1.2) | 48 (1.0) |
| Congestive heart failure | 2 (1.1) | 9 (1.0) | 17 (1.7) | 36 (0.8) |
| Lymphoma | 2 (1.1) | 3 (0.3) | 6 (0.6) | 22 (0.5) |
| Metastatic solid tumour | 2 (1.1) | 4 (0.4) | 8 (0.8) | 17 (0.4) |
| Dementia | 0 (0) | 3 (0.3) | 2 (0.2) | 16 (0.3) |
| Hemiplegia | 0 (0) | 2 (0.2) | 0 (0) | 4 (0.1) |
| Leukemia | 0 (0) | 1 (0.1) | 2 (0.2) | 5 (0.1) |
| AIDS | 0 (0) | 0 (0) | 0 (0) | 3 (0.1) |
